# Pharmacological evaluation of tacrolimus (FK-506) on ischemia reperfusion induced vasculatic neuropathic pain in rats

**DOI:** 10.1186/1749-7221-5-13

**Published:** 2010-06-07

**Authors:** Arunachalam Muthuraman, Shailja Sood

**Affiliations:** 1 Rayat institute of pharmacy, Ropar campus, Nawanshahr district, Railmajra, Near Ropar-144533, Punjab, India

## Abstract

**Background:**

Ischemia reperfusion (I/R) is common in various pathological conditions like diabetic complication, rheumatic arthritis, necrotizing vascular occlusive disease and trauma.

**Methods:**

We have evaluated the effect of tacrolimus (1, 2 and 3 mg/kg, *p.o*. for 10 consecutive days) on femoral arterial ischemic reperfusion (I/R) induced neuropathic pain in rats. Behavioral parameters (i.e. hot plate, radiant heat, acetone drop, tail heat hyperalgesia, tail flick and tail cold allodynia tests) were assessed at different time intervals (i.e. 0, 1, 4, 7, 10, 13 and 16^th ^day) and biochemical analysis in serum and tissue samples were also performed along with histopathological studies.

**Results:**

Behavioral pain assessment revealed increase in the paw and tail withdrawal threshold in tacrolimus treated groups against hyperalgesic and allodynic stimuli as compared to the sham control group. We observed a decrease in the serum nitrate and thiobarbituric acid reactive substance (TBARS) levels along with reduction in tissue myeloperoxidase (MPO) and total calcium levels, whereas, rise in tissue reduced glutathione levels in tacrolimus treated groups. However, significant results were obtained in medium and high dose treated group as compared to sham control group. Histopathological study had revealed the increase in the neuronal edema and axonal degeneration in the I/R group whereas, tacrolimus ameliorate these effects.

**Conclusion:**

Our results indicate the anti-oxidative, anti-inflammatory and calcium modulatory actions of tacrolimus. Therefore, it can be used as a therapeutic agent for the treatment of vascular inflammatory related neuropathic pain.

## Introduction

Clinically, neuropathic pain is characterized by sensory symptoms, impairment of motor function as well as vasomotor and sudomotor abnormalities that typically show a spreading tendency with a generalized distal distribution [1]. The peripheral mechanism discussed above include immune cell mediated inflammatory process [2,3], autoimmune inflammatory process [4], neurogenic inflammation [3,5] and tissue hypoxia [6]. However, according to central mechanism develops as a consequence of reorganization of somatosensory, somatomotor and autonomic systems in the CNS triggered by a peripheral input [7].

Novel neuropathic pain model has been proposed in complex regional pain syndrome (CRPS) produced by prolonged hindpaw ischemia and reperfusion in rat [8]. Ischemic-reperfusion event is well documented to induce potent injury in the targeted organs, which were indicated in the myocardial, renal, liver, lung, stomach and neuronal cells [9-11]. Ischemic-reperfusion process leads to change in the microvascular environment which in turn causes neuronal edema, breakdown of blood-nerve barrier, nerve fiber degeneration, neuronal excitation, decreased nerve conduction velocity, membranous lipid peroxidation, accumulation of free radical, alteration of enzymatic reaction, ion fluxes etc [12,13].

This alteration in neuronal blood flow and neuronal function may leads to partial and/or permanent impairment of quality of life in neuropathic patients. Certain pathological conditions are responsible for the development of vasculatic neuropathy such as diabetes mellitus, vascular occlusive diseases, necrotizing vasculitides, peripheral arterial disease, trauma etc [14]. Moreover, peripheral vascular changes are common progressive factors for the acute and chronic ischemic neuropathic pain in patients [15].

The pathophysiology of I/R injury include platelet aggregation, immune cell activation, free radical generation and leukocyte-endothelial cell interactions which lead to the injury of the endothelium and obstruction of capillaries, thus impairing oxygen supply to the nerve tissue [16]. Tacrolimus (FK506) is a potent immunosuppressive drug that has been widely used for organ transplantation and atopic dermatitis. Recently, clinical studies have demonstrated the beneficial effects of this agent in the treatment of various autoimmune and inflammatory diseases such as, rheumatoid arthritis and inflammatory bowel diseases [17]. Tacrolimus has also been reported to possess ameliorative role in the peptic ulcer due to its antioxidant and immunosuppressive action [18]. Therefore, the present study was designed to investigate the ameliorative effect of FK-506 (tacrolimus) on femoral ischemia-reperfusion injury induced neuropathic pain in rats.

## Materials and methods

### Animal

Wistar rats of either sex weighing between 180-250 g were used. Animals were procured from Punjab Agriculture University, Department of Animal Sciences, Ludhiana. They were kept at standard laboratory diet, environmental temperature and humidity. A 12 h natural light and dark cycle was maintained throughout the experimental protocol. The animals had free access to standard laboratory chow and water *ad libitum*. The experimental protocol was duly approved by Institutional Animal Ethics Committee (IAEC) and care of the animals was carried out as per the guidelines of Committee for the Purpose of Control and Supervision of Experiments on Animals (CPCSEA), Ministry of Environment and Forest, Government of India (Reg No:- 874/ac/05/CPCSEA).

### Chemicals

DTNB (5,5'-dithio bis (2-nitrobenzoic acid), BSA (Bovine Serum Albumin), (GSH) reduced glutathione were purchased from Sisco Research Laboratories, Mumbai. Thiobarbituric acid was purchased from Loba Chemie, Mumbai. All other reagents were obtained from S.D. Fine Chemicals, Mumbai, India.

### Surgical procedure

Rats were anesthetizsed intraperitoneally with ketamine HCl (50 mg/kg) and xylazine (5 mg/kg). Animals were then placed in supine position on a heated mat during the operation and recovery. Right femoral vessels were exposed through an inguinal incision and were dissected free from the femoral nerve under operating microscope. Near the trifurcation of the sciatic nerve (into peroneal, tibial and sural branches) ischemia was developed for three hours by occluding the femoral artery with a silk suture (6-0) using slipknot technique [19] and later on reperfusion was achieved by the removal of this ligature. Venous and femoral nerve occlusion was carefully avoided. To prevent thrombosis of the artery, two subcutaneous injections of heparin (8 IU, Roche in 0.3 ml saline) were given, one at the beginning and one at the end of the period of ischemia. In all the groups, silk suture was removed after 3 h ischemic event to allow reperfusion up to 21 days study protocol. Blood flow was checked under a microscope at the distal site of ligature after removing the silk thread. The animals were placed under heating lamps until they recovered from anesthesia.

#### Behavioral Study

##### Hot plate test

Thermal nociceptive threshold, as an index of thermal-hyperalgesia, was assessed by the hot plate test as described by Andreas and Rainer [20]. Eddy's hot plate was pre-heated and maintained at temperature of 52.5 ± 0.5°C. Rats were placed on the hot plate and nociceptive threshold was assessed with respect to hind paw licking. Response was recorded in seconds. Cut-off time of 20 s was maintained.

##### Plantar test

Radiant heat sensitivity of right hind paw was measured under the radiant heat lamp source as described by Hargreaves *et al*., [21]. The intensity of the radiant heat stimulus was maintained at 25 ± 0.1°C. Response of paw withdrawal latency was noted in seconds. Cut-off time of 15 s was maintained.

##### Acetone drop test

Thermal (non-noxious cold) non-nociceptive threshold, as an index of cold allodynia, was assessed by using acetone drop method as described by Choi *et al*., [22]. The reactivity to non-noxious cold chemical stimuli was assessed. Rat was placed on the top of the wire mesh grid, allowing access to the hind paws. Acetone (100 μl) was sprayed on the plantar surface of the hind paw of rat and time taken to appear the cold sensitive reaction with respect to either paw licking, shaking or rubbing the hind paw was recorded within 20 seconds.

##### Tail heat hyperalgesia test

Spinal thermal sensitivity was assessed by the tail immersion test as described by Necker and Hellon [23]. Tail heat-hyperalgesia was noted with the immersion of terminal part of the tail (1 cm) in water, temperature was maintained at 52.5 ± 0.5°C. Duration of the tail withdrawal reflex was recorded, as a response of spinal heat sensation and a cut-off time of 15 s was maintained.

##### Tail flick test

Spinal thermal sensitivity was assessed by the tail flick test as described by D'Amour and Smith [24]. Temperature of heating element (nichrome wire) of analgesiometer was maintained at 52 ± 0.5°C. The tail of rat was placed on analgesiometer at uniform distance from the nichrome wire. The tail flick response was noted and cut-off time of 15 s was maintained.

##### Tail cold allodynia test

Spinal thermal sensitivity was assessed by the tail immersion test as described by Necker and Hellon [23]. Briefly, the terminal part of the tail (1 cm) of the rat was immersed in cold non-noxious temperature (8 ± 0.5°C), until the tail was withdrawn. The duration of the tail withdrawal reflex was recorded and a cut-off time of 20 s was used.

#### Biochemical study

Blood samples were collected by retro-orbital sinus puncture at different day's interval (i.e., day 0, 4, 8, 12, and 16^th^). Serum samples were prepared for the evaluation of oxidative stress marker (nitrate and TBARS) changes in rats. Further, tissue samples were employed to estimate reduced glutathione, total calcium, MPO and histopathological evaluation.

##### Estimation of serum nitrate level

The oxidized end product of NO i.e. nitrate was measured in serum samples using a procedure based on the Griess reaction [25]. Potassium nitrate (80 mM) was used as a standard for the determination of nitrate. Serum nitrate levels were expressed as μmol/L.

##### Estimation of lipid peroxidation (TBARS)

Serum malondialdahyde (MDA) level, an index of lipid peroxidation, was determined by thiobarbituric acid (TBA) reaction. The principle of the method depends on measurement of the pink color produced by interaction of barbituric acid with malondialdahyde. 1,1,3,3-tetraethoxypropane was used as a primary standard. The determination of MDA level was performed by the method of Yagi [26]. Serum MDA levels were expressed as nmol/ml.

##### Estimation of total protein content

Protein concentration was estimated according to the method of Lowry *et al*., [27] using bovine serum albumin as a standard. The absorbance was determined spectrophotometrically at 750 nm.

##### Estimation of reduced glutathione

Reduced glutathione levels were estimated according to the method of Ellman [28]. Equal quantity of tissue homogenate was mixed with 10% trichloroacetic acid and centrifuged to separate out protein. To 0.01 ml of this supernatant, 2 ml of phosphate buffer (pH 8.4), 0.5 ml of 5,5'-dithio, bis(2-nitrobenzoic acid) and 0.4 ml of double distilled water was added. Mixture was vortexed and the absorbance was taken at 412 nm within 15 min. The concentration of reduced glutathione was expressed as μmol/g of protein.

##### Estimation of total calcium

Total calcium levels were estimated in the sciatic nerve as described by Severnghaus and Ferrebee [29] and Muthuraman *et al*., [12]. Briefly, the sciatic nerve homogenate was mixed with 1 mL of trichloroacetic acid (4%) in the ice-cold condition and centrifuged at 1500 × *g *for 10 min. The clear supernatant was used for estimating the total calcium levels by atomic emission spectroscopy at 556 nm.

##### Estimation of myeloperoxidase activity

MPO, an enzyme liberated due to activation of polymorphonuclear leukocytes, is used as an indication of tissue neutrophil accumulation. MPO activity was measured using a procedure similar to that documented by Hillegass *et al*., [30]. Sciatic nerve tissues were homogenized in 50 mM potassium phosphate buffer (pH 6.0), and centrifuged at 2500 rpm (10 min); pellets were resuspended in 50 mM phosphate buffer containing 0.5% hexadecyltrimethylammoniumbromide (HETAB). After three freeze and thaw cycles, with sonication between cycles, the samples were centrifuged at 2500 rpm for 10 min. Aliquots (0.3 ml) were added to 2.3 ml of reaction mixture containing 50 mM phosphate buffer, o-dianisidine, and 20 mmol H_2_O_2 _solution. The presence of MPO was measured at 460 nm for 3 minutes. MPO activity was expressed as U per g tissue. One unit of MPO activity was defined as that degrading 1 μmol peroxide per min at 25°C.

#### Histopathological study

##### Assessment of axonal degeneration

Samples of sciatic nerve were stored in the fixative solution (10% formalin) and cut into 4 μm thickness size. Staining was done by using hematoxylin and eosin as described by Yukari *et al*., [31]. Nerve sections were analyzed qualitatively under light microscope (450 ×) for axonal degeneration.

##### Experimental Design

Seven groups were employed in the present study, each consist of six Wistar rats.

###### Group I (Normal control group)

Rats were not subjected to any surgical procedure and were kept for 21 days. Behavioral tests were employed to assess nociceptive threshold on day 0, 1, 4, 7, 10, 13 and 16^st ^whereas, biochemical analysis was performed for the estimation of serum nitrate and TBARS on day i.e., day 0, 4, 8, 12, and 16, all animals were sacrificed by cervical dislocation and sciatic nerve tissues were immediately isolated for the study of biochemical (reduced glutathione, total calcium and MPO) and histopathological changes.

###### Group II - Sham control group

Rats were subjected to surgical procedure to expose right femoral artery without any vascular damage and ischemia. Behavioral and biochemical tests were employed on different days as described in group I.

###### Group III - Ischemia-reperfusion control group [I/R]

Rats were subjected to surgical procedure to expose and develop 3 h ischemia followed by prolong reperfusion on right femoral artery. Behavioral tests and biochemical parameters were assessed as described in group I.

###### Group IV - Vehicle treated group [I/R + Vehicle]

Vehicle (1% CMC *p.o*.) was administered to all the rats upto the end of the study protocol. Behavioral tests and biochemical parameters were assessed as described in group I.

###### Group V to VII - FK-506 treated group [I/R + FK-506 (1, 2 and 3 mg/kg)]

FK-506 (1, 2 and 3 mg/kg, *p.o*.) doses were administered in group V to VII respectively upto the end of the study protocol. Behavioral tests and biochemical parameters were assessed as described in group I.

##### Statistical Analysis

All the results were expressed as mean ± standard error of means (S.E.M). Data obtained from behavioral and serum biochemical tests were statistically analyzed using two-way analysis of variance (ANOVA). The data of tissue biomarker total calcium and MPO were analyzed using one way analysis of variance (ANOVA). In both cases, Tukey's multiple range tests were applied for *post-hoc *analysis by using Graph pad prism Version-5.0 software. A probability value of *p *< 0.05 was considered to be statistically significant.

## Results

### Behavioral study

**Figure 1:**
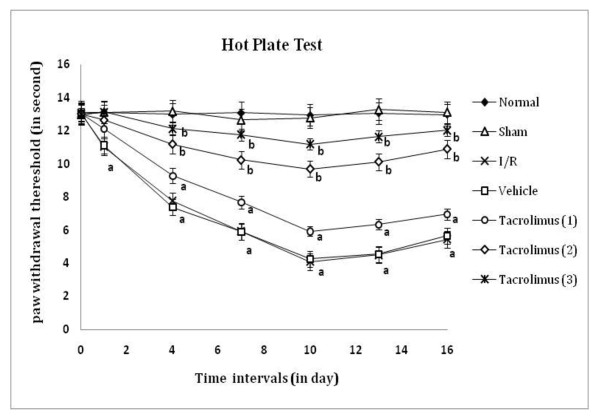
**Time course of paw thermal hyperalgesia was measured against noxious conduct heat evoked hind paw licking response**. Data were expressed as mean ± S.E.M., n = 6 rats per group. **a **= *p *< 0.05 vs sham control group, **b **= *p *< 0.05 vs I/R control group.

**Figure 2:**
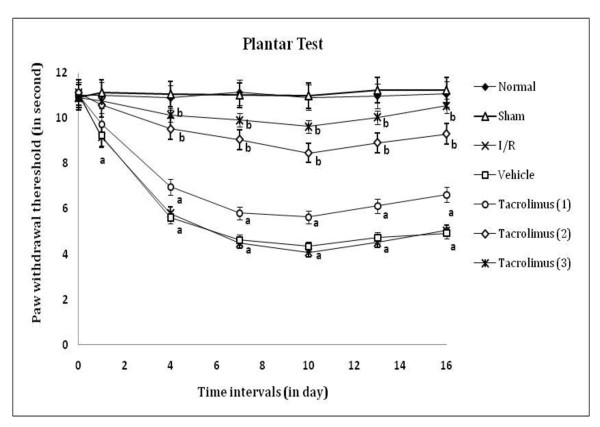
**Time course of peripheral thermal hyperalgesia was measured against noxious radiant heat evoked ipsilateral right hind paw withdrawal response**. Data were expressed as mean ± S.E.M., n = 6 rats per group. **a **= *p *< 0.05 vs sham control group, **b **= *p *< 0.05 vs I/R control group.

**Figure 3:**
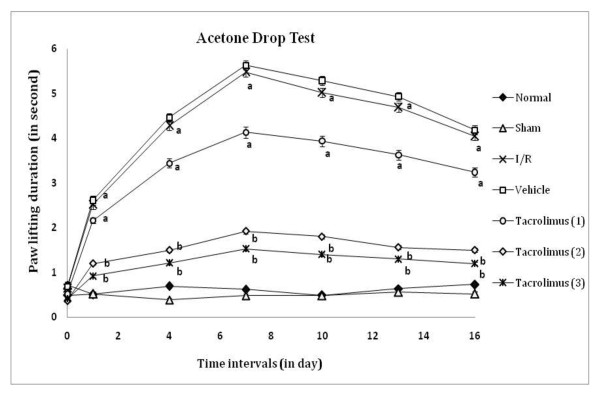
**Time course of paw cold allodynia was measured against non-noxious chemical cold evoked paw withdrawal response**. Data were expressed as mean ± S.E.M., n = 6 rats per group. **a **= *p *< 0.05 vs sham control group, **b **= *p *< 0.05 vs I/R control group.

Peripheral thermal (conduction, radiant and chemical) sensitivity was assessed by paw withdrawal threshold and paw lifting duration, as an index of heat hyperalgesia and chemical allodynia by using hot plate, radiant heat lamp and acetone applicator respectively as shown in figure 1, 2 and 3. I/R of femoral artery showed significant decrease in paw withdrawal threshold and increase in paw lifting duration at different days with maximum effect shown at 7^th ^day as compared to sham control group. Whereas, tacrolimus treated groups V to VII showed increase in paw withdrawal threshold and decrease in paw lifting duration but significant results were observed only in the medium and high dose (2 and 3 mg/kg, p.o.) treated groups as compared to I/R control group.

**Figure 4:**
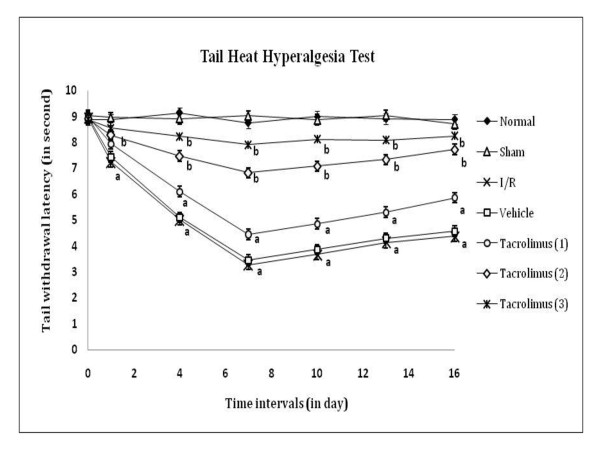
**Time course of tail thermal hyperalgesia was measured against noxious warm water immersion evoked tail withdrawal response**. Data were expressed as mean ± S.E.M., n = 6 rats per group. **a **= *p *< 0.05 vs sham control group, **b **= *p *< 0.05 vs I/R control group.

**Figure 5:**
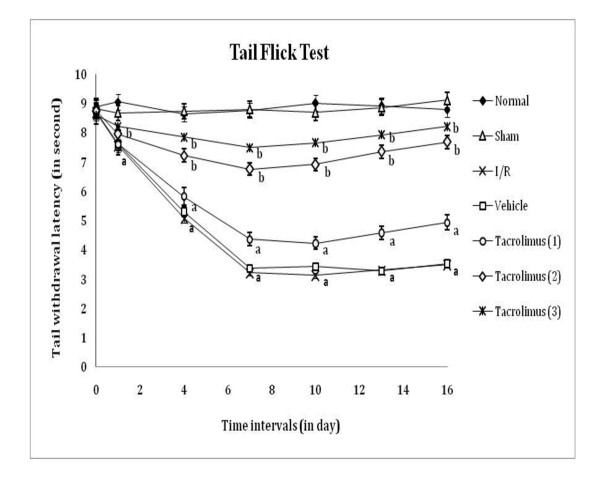
**Time course of tail thermal hyperalgesia was measured against noxious radiant heat evoked tail withdrawal response**. Data were expressed as mean ± S.E.M., n = 6 rats per group. **a **= *p *< 0.05 vs sham control group, **b **= *p *< 0.05 vs I/R control group.

**Figure 6:**
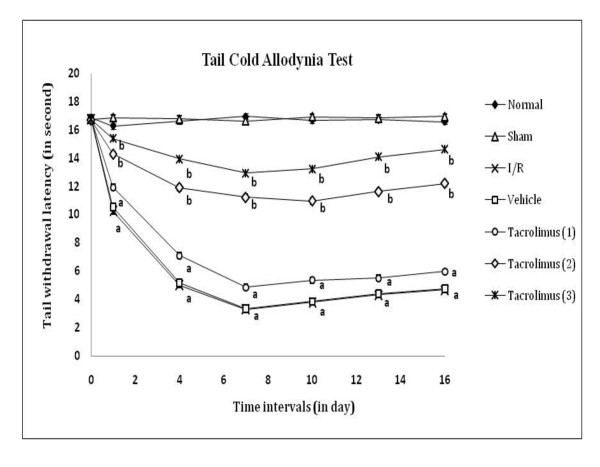
**Time course of tail thermal allodynia was measured against non-noxious cold water immesion evoked tail withdrawal response**. Data were expressed as mean ± S.E.M., n = 6 rats per group. **a **= *p *< 0.05 vs sham control group, **b **= *p *< 0.05 vs I/R control group.

Spinal thermal (conduction and radiant) and cold sensitivity were assessed by tail withdrawal latency, as an index of heat hyperalgesia and cold allodynia by using hot water (52 ± 0.5°C), analgesiometer and cold water (8 ± 0.5°C) respectively as shown in figure 4, 5 and 6. I/R of femoral artery showed significant decrease in tail withdrawal latency at different days with maximum effect shown at 7^th ^day as compared to sham control group. Whereas, tacrolimus treated groups V to VII showed increase in tail withdrawal latency but significant results were observed only in the medium and high dose (2 and 3 mg/kg, *p.o*.) treated groups as compared to I/R control group.

### Biochemical study

I/R control group had shown increase in serum nitrate and TBARS levels as compared to sham control group at different day's interval. Further, sciatic nerve tissue samples also showed significant changes in biochemical parameters i.e. increased total calcium level and MPO activity but decreased reduced glutathione level as compared to sham control group. However, tacrolimus treated groups V to VII showed ameliorative effect on serum and tissue biomarker changes but significant results were observed only in the medium and high dose (2 and 3 mg/kg, p.o.) treated groups as compared to I/R control group (Table 1 and 2).

**Table 1:** Effect of tacrolimus on I/R induced changes in serum nitrate and MDA level

Nitrate level (μmol/l)	Groups	0 day	4^th ^day	8^th ^day	12^th ^day	16^th ^day
	
	Normal	19.24 ± 0.31	19.91 ± 0.28	19.58 ± 0.42	19. 73 ± 0.34	19.92 ± 0.49
	Sham	20. 79 ± 0.39	19.57 ± 0.36	19.87 ± 0.47	19.62 ± 0.32	19.35 ± 0.27
	I/R	20.16 ± 0.26	31.43 ± 1.12^a^	36.27 ± 1.08^a^	31.27 ± 0.79^a^	29.82 ± 0. 74^a^
	Vehicle	20.36 ± 0.39	31.26 ± 1.13^a^	38.16 ± 1.09^a^	32.53 ± 0.83^a^	28.93 ± 0.59^a^
	Tacrolimus (1)	20.97 ± 0.28	36.67 ± 1.38^a^	42.94 ± 1.46^a^	37.39 ± 0.83^a^	34.69 ± 1.25^a^
	Tacrolimus (2)	20.62 ± 0.64	43.78 ± 0.64^b^	49.38 ± 0.46^b^	44.67 ± 0.46^b^	41.67 ± 0.32^b^
	Tacrolimus (3)	20.09 ± 0.42	49.59 ± 0.54^b^	57.35 ± 0.36^b^	54.56 ± 0.78^b^	51. 74 ± 0.34^b^
**MDA level (nmol/l)**	Normal	0.81 ± 0.32	0.83 ± 0.46	0. 79 ± 0.31	0.82 ± 0.36	0.83 ± 0.43
	Sham	0.82 ± 0.46	0.81 ± 0.32	0.83 ± 0.31	0.81 ± 0.34	0.85 ± 0.39
	I/R	0.74 ± 0.39	18.94 ± 1.74^a^	24.54 ± 1.56^a^	18.31 ± 1.58^a^	16.42 ± 2.23^a^
	Vehicle	0.86 ± 0.23	18.61 ± 1.34^a^	25.59 ± 1.61^a^	17.39 ± 1.57^a^	17.14 ± 2.01^a^
	Tacrolimus (1)	0.82 ± 0.38	26.43 ± 1.58^a^	33.43 ± 1.67^a^	28.81 ± 1.58^a^	26. 74 ± 1.32^a^
	Tacrolimus (2)	0.84 ± 0.36	40.36 ± 1.34^b^	44.61 ± 1.25^b^	37.67 ± 1.46^b^	34.38 ± 1.52^b^
	Tacrolimus (3)	0.81 ± 0.29	49.71 ± 1.45^b^	56.36 ± 1.54^b^	49.69 ± 1.39^b^	46.41 ± 1.23^b^

Data were expressed as mean ± S.E.M. for each group. **a **= *P *< 0.05 vs sham control group, **b **= *P *< 0.05 vs ischemia control group.

**Table 2:** Effect of tacrolimus on I/R induced changes in tissue biomarker level

Groups	Reduced Glutathione (μg/mg of protein)	MPO Activity (U/min/mg of protein)	Total Calcium (ppm/mg of protein)
Normal	72.64 ± 2.91	11.32 ± 1.56	3.49 ± 1.04
Sham	71.31 ± 2.47	12.64 ± 1.37	3.59 ± 0.83
I/R	39.25 ± 1.67^a^	134.01 ± 3.91^a^	34.61 ± 1.93^a^
Vehicle	40.54 ± 1.92^a^	138.43 ± 3.51^a^	35.19 ± 1.64^a^
Tacrolimus (1)	46.47 ± 2.65^a^	126.32 ± 1.47^a^	31.69 ± 0.57^a^
Tacrolimus (2)	63.35 ± 1.69^b^	62.79 ± 2.63^b^	14.41 ± 2.61^b^
Tacrolimus (3)	68.41 ± 1.83^b^	41.84 ± 3.92^b^	9.16 ± 1.92^b^

Data were expressed as mean ± S.E.M. for each group.

**a **= *P *< 0.05 vs sham control group,

**b **= *P *< 0.05 vs ischemia control group.

### Histopathological study

**Figure 7:**
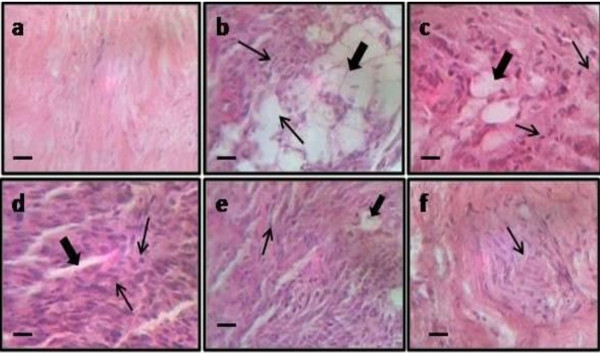
**Effect of femoral artery I/R induced neuronal histopathological changes shown in figure a to f (sham, ischemia control, vehicle, tacrolimus (1), tacrolimus (2) and tacrolimus (3) respectively)**. Fig. b shows neuronal edema and degeneration as compared to sham control group. Moreover, fig e and f shows amelioration of tacrolimus (2 and 3 mg/kg) on neuronal edema and degeneration in sciatic nerve of rat. Microscopic examinations were performed under 450 × light microcopy, scale bar 10 μm.

I/R injury of femoral artery resulted in significant histopathological changes which were assessed in cross sectional section of distal part of sciatic nerve. In cross section, axonal degeneration was shown by decrease in number of myelinated fibers along with swelling of non-myelinated and myelinated nerve fibers. But tacrolimus treatment (2 and 3 mg/kg) resulted in attenuation of I/R induced axonal degeneration and histopathological alterations (Fig. 7).

## Discussion

In the present study, tacrolimus showed significant amelioration of ischemia reperfusion induced behavioral, biochemical and histopathological changes. Literature revealed that ischemia followed by reperfusion can cause severe damage in heart, intestine, kidney, stomach, brain and peripheral nerve [32]. Ischemic insult of vascular and nervous system in vascular occlusive diseases, necrotizing vasculitides, diabetes mellitus and trauma plays a major key role in the development of ischemic pain, vasculatic neuropathic pain etc [33,14]. Severe ischemic insult in nerve has resulted in the energy shutdown followed by conduction failure and fiber degeneration [19]. The most important hypothesis explains that the neuronal cellular reperfusion induced damage is caused by enhancement of the free radical generation, lipid peroxidation, calcium overload, alteration in the level of nitrite/nitrate, pro/anti-inflammatory cytokines and neuronal apoptotic components, endoneurial edema and augmentation of fiber degeneration [34]. Both ischemic insult and reperfusion process can alter the structural and functional action of the certain targeted cells. In the present study the peripheral nerve has been targeted for induction of vasculatic neuropathy in rats by the process of femoral artery I/R. The event of femoral artery I/R process has been well documented for the induction of the neuro-inflammation, neuronal excitability and enhancement of pain sensation [35].

The production of reactive oxygen species and reactive nitrogen species (ROS/RNS) in severe oxidative stress conditions such as sepsis, trauma, surgery, ischemia, hypoxia and ischemia-reperfusion lead to the loss of membrane integrity and structural or functional changes [36]. Further, generation of free radicals can cause neuronal and endothelial damage through the induction of lipid peroxidation, protein oxidation and direct damage to nucleic acids [37]. Nitric oxide (NO) is an important endogenous vasodilator in the vascular system and plays a protective role in the cardiovascular and other vital organ system. In contrast, it has been suggested that the neuronal blood flow is maintained at low concentration of NO and the excessive release of NO may be toxic to the nerve cells [38]. This toxicity may be exacerbated during ischemia and reperfusion due to generation of O_2 _leading to formation of the peroxynitrite radicals [39]. In the present study, the effect of I/R induced behavioral changes were assessed by the hot plate, plantar, acetone drop, tail (heat and cold water) immersion and tail flick tests. Further, neuro-vascular changes were evaluated by direct measurement of the level of nitrate and TBARS in serum and tissue reduced glutathione, total calcium and MPO activity. Results obtained had confirmed I/R injury induced vasculatic neuropathy in rats. However, tacrolimus treatment had resulted in the reduction of such neuropathic pain along with ameliorative effect on biochemical parameters and such I/R induced vasculatic neuropathy clinically resemble to diabetic, rheumatoid vasculatitis, vascular inflammatory and demyelinating related neuropathy [40].

Ischemia reperfusion induced vasculitic neuropathy has shown compelling evidence for the role of myeloperoxidase due to mast cell activation. The pathogenesis of vasculitis is complex and is the result of various autoimmune reactions, both humoral and cell mediated. There are multiple triggering events or antigens leading to various immunological and histological responses [41]. Moreover, free radicals are also found to be involved in chronic constriction injury, tibial sural transection, axotomy, traumatic injury and peripheral ischemia reperfusion induced neuropathic pain [6,12,13]. Peripheral ischemia is recognized as a secondary phenomenon in patients with peripheral arterial disease, vasculatic neuropathy etc. Obstruction of the peripheral arteries of the legs develop peripheral nerve dysfunctions including peripheral ischemic pain in the lower limbs which may be due to the free radicals generation, immune cell activation, calpain activation etc [42].

It is well known that tacrolimus (FK-506) inhibit the induction of iNOS by suppressing the activation of nuclear factor kappa-B (NF-κB) [43]. Recently, it has also been reported that the anti-oxidative, anti-inflammatory and calcium modulatory actions of tacrolimus prevented gastric mucosal lesions [18]. Results revealed that tacrolimus reduce serum nitrate and TBARS levels along with reduction in the tissue total calcium and MPO activity but it showed increase in tissue reduced gluthathion levels. Therefore, from the above discussion it may be concluded that these ameliorative effects on various biomarkers may be due to its effect on decrease in free radical accumulation and inflammatory markers as well as its calcium modulatory actions [18,44].

Histopathological evaluation had also revealed I/R induced axonal degeneration. In fact in I/R induced axonal degeneration, calcium influx has been considered as one of the early events following axon injury that signals the resealing of the severed end by a vesicle mediated process. Calcium induced activation of calpains has been reported in the axonal degeneration [12,13]. Calcium induced activation of calpain is also associated with generation of reactive oxygen species from mitochondria [45]. Therefore, tacrolimus prevented the axonal degeneration may be due to its calcenurin inhibitor activity.

## Conclusion

Hence, it may be concluded that tacrolimus may act as potential agent for the amelioration of ischemia reperfusion induced neuropathic pain (complex regional pain syndrome) due to its antioxidant, calpain inactivation and immunosuppressive actions.

## Competing interests

The authors declare that they have no competing interests.

## Authors' contributions

AM and SS performed experiment procedure, surgery and evaluation of behavioral, biochemical and histopathological study. The authors read and approved the final manuscript.
